# Stability of *Lactobacillus rhamnosus* GG in prebiotic edible films

**DOI:** 10.1016/j.foodchem.2014.03.008

**Published:** 2014-09-15

**Authors:** Christos Soukoulis, Solmaz Behboudi-Jobbehdar, Lina Yonekura, Christopher Parmenter, Ian D. Fisk

**Affiliations:** a Division of Food Sciences, School of Biosciences, University of Nottingham, Sutton Bonington Campus, Loughborough LE12 5RD, Leicestershire, United Kingdom; b Nottingham Nanotechnology and Nanoscience Centre, University of Nottingham, University Park, Nottingham NG7 2RD, Nottinghamshire, United Kingdom

**Keywords:** Edible films, Probiotics, Synbiotics, Survival, Encapsulation

## Abstract

The concept of prebiotic edible films as effective vehicles for encapsulating probiotic living cells is presented. Four soluble fibres (inulin, polydextrose, glucose-oligosaccharides and wheat dextrin) were selected as prebiotic co-components of gelatine based matrices plasticised with glycerol and used for the immobilisation of *Lactobacillusrhamnosus* GG. The addition of prebiotics was associated with a more compact and uniform film structure, with no detectable interspaces or micropores; probiotic inclusion did not significantly change the structure of the films. Glucose-oligosaccharides and polydextrose significantly enhanced *L. rhamnosus* GG viability during air drying (by 300% and 75%, respectively), whilst a 33% and 80% reduction in viable counts was observed for inulin and wheat dextrin. Contrarily, inulin was the most effective at controlling the sub-lethal effects on *L. rhamnosus* GG during storage. However, in all cases the supplementation of edible films with prebiotics ameliorated the storage stability of *L. rhamnosus* GG.

## Highlights

• The concept of prebiotic gelatine based edible films containing probiotics is presented.
• Prebiotic edible films effectively protected *L. rhamnosus* GG.
• Inulin and wheat fibre improved the storage stability of *L. rhamnosus* GG.
• Glucose-oligosaccharides and polydextrose reduced lethality during air drying.
• Prebiotics resulted in a more compact, less porous and reticular film structure.

## 1 Introduction

According to (FAO/WHO, 2002) the term probiotics is used to define “viable organisms which when administered in adequate amount (10^6^ to 10^7^ CFU/g) to the human host confer health benefits”. Delivering probiotics through ingestion of functional foods has been proposed to be associated with several health benefits including regulation of the gastro-intestinal tract, stimulation of the immune system, reduction of serum cholesterol levels, relief of lactose intolerance and irritable bowel syndrome symptomatology, prevention of cardiovascular disease and several forms of cancer (Chong, 2014; Kumar et al., 2010; Saad, Delattre, Urdaci, Schmitter, & Bressollier, 2013). Incorporation of probiotics in real food matrices is rather challenging due to the wide range of detrimental processes that take place due to food processing and storage practises. For instance, probiotic living cells are subjected to osmotic, heat and acid induced stresses and mechanical injuries (Fu & Chen, 2011). Encapsulation of probiotic cells in low moisture (spray or freeze dried matrices), cross-linked or self-assembled biopolymer microparticulates and recently immobilisation in single or composite biopolymer substrates e.g. edible films, are currently the commonest strategies to surpass the obstacles relating to probiotics lethality due to food processing (Anal & Singh, 2007; Cook, Tzortzis, Charalampopoulos, & Khutoryanskiy, 2012; Kanmani & Lim, 2013; López De Lacey, López-Caballero, Gómez-Estaca, Gómez-Guillén, & Montero, 2012; Soukoulis, Behboudi-Jobbehdar, Yonekura, Parmenter, & Fisk, 2013; Soukoulis et al., 2014; Yonekura, Sun, Soukoulis, & Fisk, 2014).

With respect to the industrial feasibility of probiotic edible films and coatings, a number of applications including chilled processed fruit, vegetable and fish products as well as probiotic bakery products have been developed to-date (Altamirano-Fortoul, Moreno-Terrazas, Quezada-Gallo, & Rosell, 2012; López De Lacey et al., 2012; Soukoulis et al., 2014; Tapia et al., 2007).

Prebiotics are regarded as selectively fermented ingredients that allow specific changes both in the composition and activity of the gastrointestinal microbiota which confers benefits to host well-being and health (Gibson, Probert, Van Loo, Rastall, & Roberfroid, 2004). It is well documented that the synbiotic combination of prebiotics with probiotic strains promotes colonisation in the intestinal tract inhibiting the growth of human or animal pathogens and promoting bifidogenicity (Mugambi, Musekiwa, Lombard, Young, & Blaauw, 2012). Moreover, ingestion of prebiotics has been reported as enhancing the intestinal absorption of Ca^2+^ and Mg^2+^ (bone mineralisation and lipid metabolism), as well as preventing several forms of cancer (Franck, 2002; Roberfroid, 2007b; Saad et al., 2013). In addition, prebiotics have been successfully tested as co-components for microencapsulation and in the case of anhydrobiotics (viable probiotics stabilised in a dried format) have conferred a beneficial effect on cell viability (And & Kailasapathy, 2005; Fritzen-Freire et al., 2012).

The aims of the present work were to develop and investigate several plasticised gelatine-prebiotic composite edible films containing *Lactobacillus* *rhamnosus* GG. Four oligomer carbohydrate materials with known prebiotic functionality (Roberfroid, 2007a) (inulin, polydextrose, glucose oligosaccharides and wheat dextrin) were evaluated for the first time in probiotic edible films.

## 2 Methods and materials

### 2.1 Materials

A probiotic strain (*L. rhamnosus* GG*,* E-96666, VTT culture collection, Espoo, Finland) with established probiotic functionality was used for the preparation of the edible films. Gelatine bovine skin type B, hexahydrate magnesium nitrate and glycerol (purity > 99%) were purchased from Sigma–Aldrich (Gillingham, UK). Inulin (Fibruline® S) was obtained from Cosucra SA (Wincoing, Belgium), whereas wheat dextrin (Nutriose®), polydextrose (Promitor®), and glucose-oligosaccharides (Glucofibre®) were kindly provided as a gift from Roquette, (France) and Tate & Lyle GmbH, (Germany) respectively.

### 2.2 Stock culture preparation and growth conditions of *L. rhamnosus* GG

Preparation of stock culture was carried out as described previously (Behboudi-Jobbehdar, Soukoulis, Yonekura, & Fisk, 2013). Growth of *L. rhamnosus* GG was carried out at 37 °C for 48 h under anaerobic conditions in plastic jars containing Anaerogen® (Oxoid Ltd., Basingstoke, UK). The obtained cell culture broth (found in the stationary bacterial growth stage) was aseptically transferred to sterile 50 mL plastic centrifuge tubes (Sarstedt Ltd, Leicester, UK) and centrifuged at 3000*g* for 5 min. Supernatant liquid was discarded and the harvested bacterial cells were twice washed with phosphate buffer saline (Dulbecco A, Oxoid Ltd, Basingstoke, UK).

### 2.3 Preparation of the probiotic edible films

Gelatine and prebiotic fibres (wheat dextrin, polydextrose, glucose-oligosaccharides and inulin) were dispersed in distilled water at 50 °C to obtain five individual biopolymer solutions. Glycerol was adjusted at the 40% w/w of the aliquots’ total solids. In all cases, the total solids composition of the solutions was 4% w/w of biopolymers and 1.6% w/w of glycerol. The gelatine solution was left to fully hydrate for 30 min at 50 °C, 1:1 mixed with the prebiotic solutions, and after pH adjustment at 7.0 with sodium hydroxide 0.1 M, the obtained aliquots were heat treated at 80 °C for 15 min to destroy pathogens and to fully dissolve gelatine. Then, the heated aliquots were cooled at 40 °C and kept isothermally to avoid gelatine setting until inoculation with probiotics.

Six pellets of *L. rhamnosus* GG (corresponding to 300 mL of culture broth) were added to individual film forming solutions (100 mL) and degassed using a vacuum pump at 40 °C for 10 min. Then, 30 mL of the solution was aseptically transferred using a serologic pipette to sterile petri dishes (inner diameter 15.6 cm; Sarstedt Ltd., Leicester, UK). The cast solutions were air dried at 37 °C for 15 h in a ventilated incubator (Sanyo Ltd., Japan) in order to obtain films that could be easily peeled off and had acceptable mechanical properties (absence of brittleness and adequate flexibility/extensibility). After drying, the probiotic edible films were peeled intact from the petri dishes and conditioned at room (25 ± 1 °C) or chilled temperature (4 ± 1 °C) under controlled relative humidity conditions (54% RH) in desiccators containing saturated magnesium nitrate solution.

### 2.4 Enumeration of *L. rhamnosus* GG

One mL of the probiotic film forming solution was suspended in sterile PBS and vortexed for 30 s to ensure adequate mixing using the method described by Lopéz de Lacey et al. (2012) with minor modifications. More specifically, individual 1 g film samples containing *L. rhamnosus* GG were transferred to 9 mL of sterile PBS and left to hydrate and dissolve under constant agitation in an orbital incubator at 37 °C for 1 h. The complete dissolution of the edible films had been previously been tested using edible films without probiotics and no residual insoluble material could be identified. In both cases, the resulting solutions were subjected to serial dilutions using phosphate buffer saline. Each dilution was pour plated on a MRS agar (MRS Agar, Oxoid Ltd., Basingstoke, UK) and the plates were stored at 37 °C for 72 h under anaerobic conditions to allow colonies to grow. Enumeration of the bacteria on agar plates was performed in triplicates by colony counting (Champagne, Ross, Saarela, Hansen, & Charalampopoulos, 2011) and the total counts of the viable bacteria were expressed as log colony forming units per gram (log CFU/g, CFU/g = CFU/plate × dilution factor).

The survival rate of the bacteria throughout the film forming solution drying process was calculated according to the following equation:

(1) $$\%\;\text{viability}=100\times \frac{N}{N_{0}}$$

where *N*_0_, *N* represent the number of viable bacteria prior and after the implemented drying process (Behboudi-Jobbehdar et al., 2013).

*L. rhamnosus* GG inactivation upon storage data was expressed as the value of the relative viability fraction *N*/*N*_0_. The viability data were fitted to a first order reaction kinetics model as described by the formula:

(2) $$N_{t}/N_{0}=1-k_{T}t$$

where *N*_0_ represents the initial number of the viable bacteria and *N_t_* the number of viable bacteria after a specific time of storage (in CFU/g), *t* is the storage time (in day), and *k_T_* is the inactivation rate constant at *T* temperature (day^−1^).

### 2.5 Physicochemical, optical and colour characteristics

A digital micrometre with a sensitivity of 0.001 mm was used for the measurement of the thickness of the probiotic edible films. Eight measurements were taken from different parts of the films to ensure results consistency.

Residual water content (g/100 g of film) was calculated according to AACC method 44-1502. Samples (approx. 0.5 g) were dried at 105 °C in aluminium pans for 48 h to constant weight. Residual water content was calculated according to the formula:

(3) $$\%\;\text{residual}\;\text{water}\;\text{content}=100\times \frac{w_{i}-w_{f}}{w_{i}}$$

here *w_i_*, *w_f_* are the initial and final weight of the edible films.

Colour characteristics of the edible films were determined using a Hunterlab (Reston, USA) colourimeter. The CIELab colour scale was used to measure the *L*^∗^ (black to white), *a*^∗^ (red to green), and *b*^∗^ (yellow to blue) parameters. The total colour difference Δ*E*^∗^ between the control sample and synbiozic films was calculated according to the formula:

(4) $$\Delta E^{*}=\sqrt{{\left(\Delta L^{*}\right)}^{2}+{\left(\Delta a^{*}\right)}^{2}+{\left(\Delta b^{*}\right)}^{2}}$$

where Δ*L*^∗^, Δ*a*^∗^, Δ*b*^∗^, are the luminosity, redness and yellowness intensity difference from the control sample.

Opacity of films was determined according to the method described by Núñez-Flores et al. (2012). Film specimen were cut into rectangles (0.7 × 1.5 cm^2^) and placed carefully on the surface of plastic cuvettes. Absorbance at 550 nm was measured using a UV–VIS spectrophotometer (Jenway Ltd., UK) (calibrated using an empty cuvette as blank) and films opacity was calculated according to the formula:

(4) $$\text{Opacity}=\frac{{\text{A}}_{550}}{\text{thickness}}$$

### 2.6 Morphological characterisation

A rectangular film sample was carefully deposited onto carbon tabs (Agar Scientific, Stansted, UK) and coated with carbon (Agar turbo carbon coater) to improve conductivity. The scanning electron microscope analysis (SEM) was performed on a FEI Quanta 3D 200 dual beam focused Ion Beam Scanning Electron Microscope (FIB-SEM). The images were acquired using secondary electron imaging at an accelerating voltage of 5–15 kV.

### 2.7 Statistical analysis

Two-way ANOVA (prebiotic supplements and storage temperature as factors) followed by Duncan’s post hoc comparison was carried out for unveiling the significance of prebiotics on the survivability of *L. rhamnosus* GG during drying and storage. All analyses were performed using SPSS release 17 statistical software (SPSS Inc., USA).

## 3 Results and discussion

### 3.1 Morphological characterisation and appearance of prebiotic films

The addition of prebiotic fibre was associated with a detectable decrease (*p* < 0.05) of the transparency of the edible films compared to the exclusively gelatine containing ones (Table 1). There was slight impact of probiotic addition on the opacity of the films, but the increase was not significant (*p* > 0.05); this is in accordance with the observations of Kanmani & Lim (2013). No significant differences in the luminosity (*L*^∗^) of the films were observed, whilst wheat dextrin and inulin based films exhibited the highest (*p* < 0.05) scores for green and yellow hue colour components (*a*^∗^ and *b*^∗^). In terms of colour difference (Δ*E*^∗^), polydextrose had the lowest and wheat dextrin the highest colour divergence from films without prebiotic fibre. However, it should be noted that in all cases Δ*E*^∗^ values were lower than 3 which is considered as the threshold of human perceivable colour differences (Martínez-Cervera, Salvador, Muguerza, Moulay, & Fiszman, 2011). No effects (*p* > 0.05) of the prebiotic fibres on film thickness were observed, and thus opacity and colour differences could be primarily attributed to the presence and type of prebiotic fibres.

**Fig. 1:**
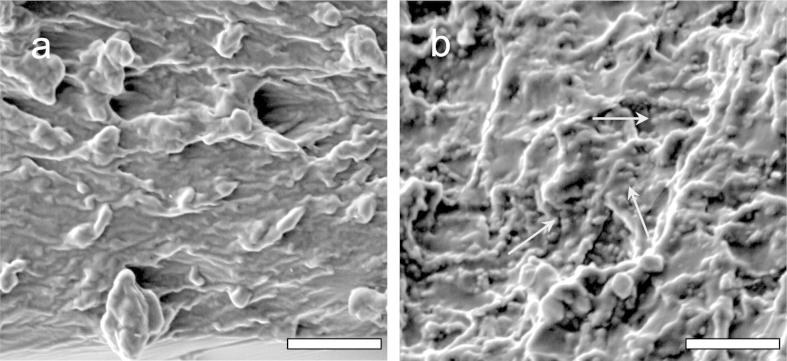
SEM visualisation of the cross-section of edible films comprised gelatine plasticised with glycerol (2:1) in the absence (a) and presence (b) of *L. rhamnosus* GG*.* Bar scale = 20 μm.

**Fig. 2:**
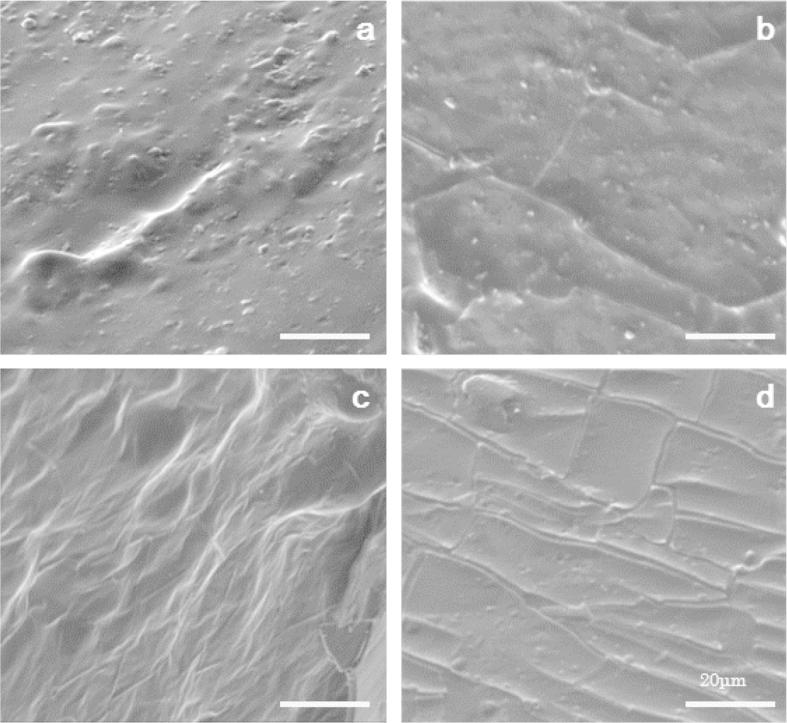
SEM visualisation of the cross-section of prebiotic gelatine edible films containing *L. rhamnosus* GG. Inulin (a), polydextrose (b), wheat dextrin (c) and glucose oligosaccharides (d). Bar scale = 20 μm.

FIB-SEM microscopic analysis of the gelatine based films allowed visualisation of *L. rhamnosus* GG cells (Fig. 1a and b). The addition of *L. rhamnosus* GG cell pellets in the edible film did not confer any noticeable modification to the structural conformation of the films (Fig. 1b), apart from the presence of the bacterial cells embedded (tiny rod-like shapes as indicated by the arrows) in the plasticised gelatine matrix. In both cases, the gelatine based films retained their cohesive, non-uniform, and reticular microstructure, as it has been also confirmed in previous studies (Jeya Shakila, Jeevithan, Varatharajakumar, Jeyasekaran, & Sukumar, 2012). The addition of prebiotics resulted in detectable changes in the microstructure of the symbiotic films (Fig. 2). As is illustrated in the SEM micrographs, blending prebiotic fibre with gelatine prior to film formation resulted to a more compact and uniform structure, with no detectable interspaces or micropores, suggesting that prebiotics act as fillers of the interspaces of entangled gelatin network. Although in all cases no bacterial cells were detected on the surface of the probiotic edible films (data not shown), cross-sectional visualisation of films unveiled enhanced coverage (and consequently better barrier properties) of the bacterial cells in the symbiotic edible films compared to those composed only of gelatine. No remarkable differences between the cross-sectional structure conformations of the films containing inulin, polydextrose and gluco-oligosaccharides were detected. It is also noteworthy that the cracks and corrugations observed in the case of polydextrose and gluco-oligosaccharides based films are related to the carbon coating and not the film structure. Films comprised wheat dextrin maintained their compact, non-porous and void-less structure, albeit more reticular and fibrous-like structure were observed. However, it should be noted, that in all cases, prebiotics exerted a good compatibility and miscibility (possibly through hydrogen bond interactions) with gelatine as no phase separation or aggregation phenomena were shown, further studies to fully characterise phase compatibility within the biopolymers were not included within this work as it was not the primary focus of the study.

### 3.2 Effect of prebiotics on *L. rhamnosus* GG throughout drying

**Fig. 3:**
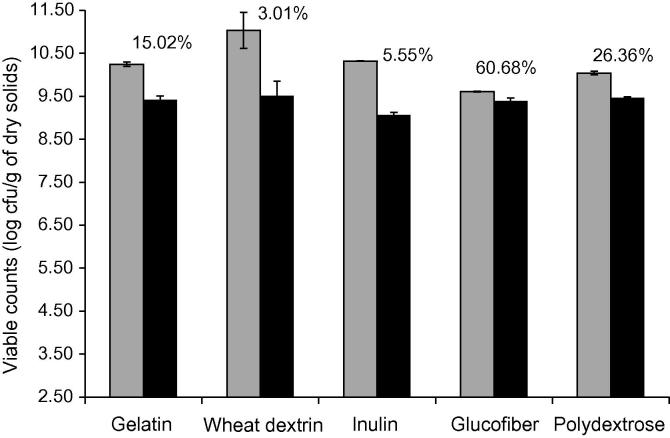
Survival of *L. rhamnosus* GG throughout air drying at 37 °C for 15 h. Viable counts are shown prior to drying (light grey) and after drying (black) with the listed% viability figure. Data are presented as mean ± SD (*n* = 3).

The viable counts of *L. rhamnosus* GG in film forming solution (start-point) and edible film (end-point) expressed on total solids basis (d.b.) are displayed in Fig. 3. The sub-lethal effects of the air drying step were found to be strongly dependent on the type of the plasticised substrate. More specifically, the addition of gluco-oligosaccharides and polydextrose provided the highest protection allowing the retention of the 60.68% and 26.36% of the initial number of living *L. rhamnosus* GG cells. A rather mediocre protection was achieved in the case of gelatine based films whilst the addition of inulin and wheat dextrin resulted into an adverse effect on cells survivability. It is well established that upon convective thermal processes, the viability of living cells is strictly influenced by both intrinsic (heat, osmotic and mechanical stress tolerance of the bacterial strains, damage of the cellular structures) and extrinsic (heat or osmotic stress pre-adaptation of the bacteria, drying kinetics and conditions, composition and structural aspects of the drying substrate, presence of thermoprotectants etc.) factors (Fu & Chen, 2011). No acute toxic effects on the viability of *L. rhamnosus* GG were observed in the film forming solutions. Moreover, viability losses due to heat induced injuries should be considered as negligible due to low drying temperatures (Ghandi, Powell, Chen, & Adhikari, 2012). By monitoring the drying kinetics (data not shown) no significant differences in the drying rates (steady and falling drying rate) and the drying time required to achieve the endpoint water activity (0.45–0.48) were detected. Thus, we presume that the detected effects on *L. rhamnosus* GG appear to be due to differences in osmotic stress. In addition, considering that during the first 4.5–5 h of drying, the water activity of the systems is higher than the critical water activity for growth of *Lactobacilli* (∼0.91), it is also presumed that the adaptation of *L. rhamnosus* GG in the drying substrate plays an important role in maintaining its biological activity. In this context, polydextrose and glucofibre can be considered as very good substrates for *L. rhamnosus* GG. Moreover, the ability of *L. rhamnosus* GG to adhere better to specific substrates has been proposed as a substantial factor for overcoming heat or osmotic induced stress with proteins being characterised by excellent adhesion properties (Burgain et al., 2013). This might be also the fact in the case of polydextrose and gluco-oligosaccharides, though further investigation is required for fully understanding the underlying mechanisms.

### 3.3 Effect of prebiotics on storage stability of *L. rhamnosus* GG

**Fig. 4:**
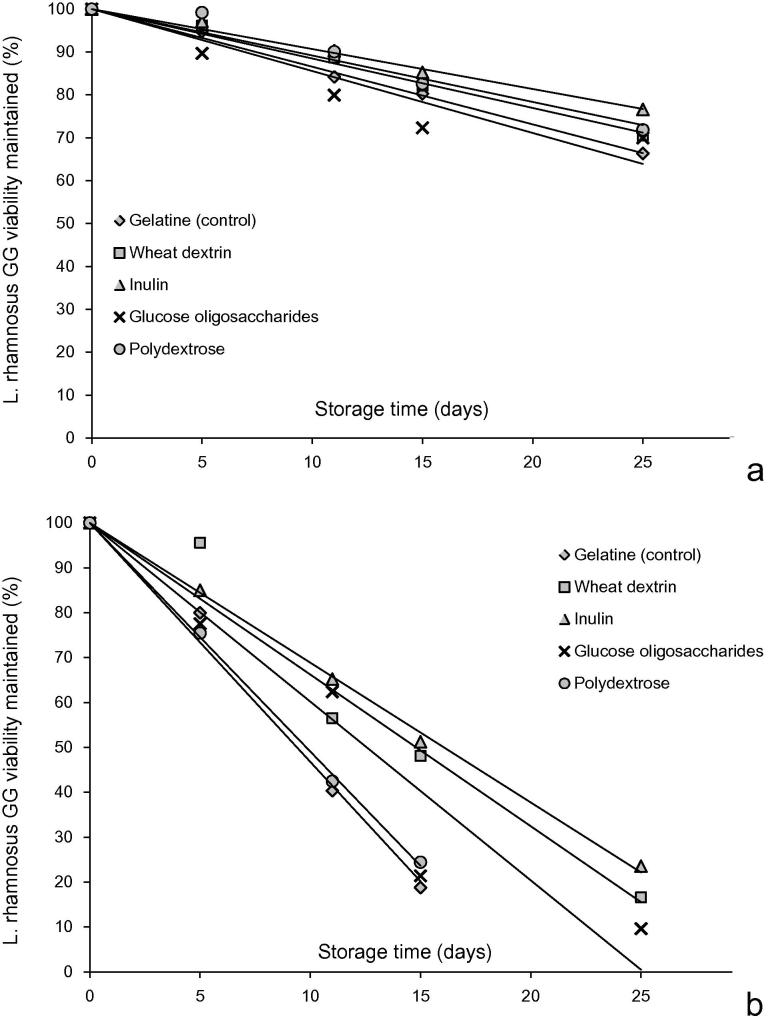
Inactivation curves of *L. rhamnosus* GG through storage at chilling (4 °C) and room (25 °C) temperature conditions for 25 days.

In Fig. 4 the inactivation curves of *L. rhamnosus* GG immobilised in edible films and stored for 25 days period at room and chilling temperature conditions are displayed. The inactivation rates (Table 1) of *L. rhamnosus* GG were, as it was expected, significantly higher (*p* < 0.001) in the systems stored at room temperature. With the exception of polydextrose edible films stored at 25 °C the presence of prebiotics in the plasticised matrices improved the storage stability of *L. rhamnosus* GG (Table 2). Inulin was the most effective fibre (based on its ability to maintain the viability of *L. rhamnosus* GG) at both storage temperatures, followed by wheat dextrin, glucose oligosaccharides and polydextrose. Increase of storage temperature induced approximately a 4-fold acceleration of the inactivation rate of *L. rhamnosus* GG, although no significant interactions between storage temperature and substrate composition were detected (*p* > 0.05). The shelf-life of the edible films (in terms of *L. rhamnosus* GG survival) ranged from 63 to 100 days and 17 to 30 days for the systems stored at chilled (4 °C) or room temperature (25 °C) conditions (Table 2). Extrinsic factors such as water activity, temperature and presence of oxygen are known to adversely influence the viability of encapsulated probiotic living cells (Fu & Chen, 2011). Moreover, the molecular mobility of solutes driven by the structural and physical state of the immobilising matrix can also influence the stability of probiotics. Thus, the acquirement of low residual water–glassy matrices with low permeability to gases containing free radical scavenging agents (to control lipid oxidation of cellular membranes) has been reported as an efficient strategy for improving probiotics viability in food systems (Dong et al., 2013; Soukoulis et al., 2013). In the case of intermediate moisture systems (including edible films) the presence of high amounts of solutes together with the rubbery physical state (solutes’ increased molecular mobility) facilitates the occurrence of enzymatic and chemical reactions that damage essential cellular structures e.g. phospholipid membrane bilayers (Fu & Chen, 2011). The stability of the prebiotic films at room temperature is generally comparable to that of anhydrobiotics (e.g. spray dried powders) stored at the same relative humidity conditions (Ying, Sun, Sanguansri, Weerakkody, & Augustin, 2012). Although a full mechanistic understanding of probiotics stability in biopolymer matrices during storage is not available, it appears that factors such as steric hindrance of solutes and the matrix translational diffusion of oxygen (both associated with the *T* − *T_g_* difference), the presence of nutrients and free radical scavenging agents as well as the interaction via hydrogen bonding with the polar head groups of membranes phospholipids can be possible explanations for the stability of probiotics in prebiotic films (Ananta, Volkert, & Knorr, 2005; Kanmani & Lim, 2013; Semyonov, Ramon, & Shimoni, 2011; Soukoulis et al., 2013). Prebiotics such as inulin, fructo-oligosaccharides and polydextrose are able to enhance the stability of probiotics primarily through their impact on the glass transition phenomena albeit no clear evidence on their specific protective action has been provided for each type of probiotics (Ananta et al., 2005; Corcoran, Ross, Fitzgerald, & Stanton, 2004; Fritzen-Freire et al., 2012). In a first attempt to provide some evidence on the impact of physical state of the matrix on the inactivation rates of *L. rhamnosus* GG, we have calculated the glass transition temperatures of the systems with the highest and lowest inactivation rate at room temperature viz. inulin and polydextrose using the Couchman–Karasz equation (Eq. (5)) assuming a quaternary system comprising fibre, gelatine, water and glycerol

(5) $$T_{g}=\frac{T_{g.\text{fibre}}{}^{*}\Delta C_{P\text{,fibre}}{}^{*}X_{\text{fibre}}+T_{g.\text{gel}}{}^{*}\Delta C_{P\text{,gel}}{}^{*}X_{\text{gel}}+T_{g\text{,glyc}}{}^{*}\Delta C_{P\text{,glyc}}{}^{*}X_{\text{glyc}}+T_{g\text{,water}}{}^{*}\Delta C_{P\text{,water}}{}^{*}X_{\text{water}}}{\Delta C_{P\text{,fibre}}{}^{*}X_{\text{fibre}}+\Delta C_{P\text{,gel}}{}^{*}X_{\text{gel}}+\Delta C_{P\text{,glyc}}{}^{*}X_{\text{glyc}}\Delta C_{P\text{,water}}{}^{*}X_{\text{water}}}$$

using literature data (Ribeiro, Zimeri, Yildiz, & Kokini, 2003; Sobral & Habitante, 2001; Zimeri & Kokini, 2002) for the glass transitions of each pure component (inulin: *Tg* = 120 °C, Δ*C_p_* = 0.65 J/gK, polydextrose: *T_g_* = 94 °C, Δ*C_p_* = 0.33 J/gK, water: *T_g_* = −139 °C, Δ*C_p_* = 1.94 J/gK, glycerol: *T_g_* = −83 °C, Δ*C_p_* = 1.25 J/gK) and mass fractions *x_i_* calculated according to the residual water content of the 54% RH conditioned films (Table 1) the glass transition temperatures for the inulin and polydextrose systems were predicted to be 15.7 and −14.63 °C, respectively. It therefore appears that phase transitions occurring due to the differing storage conditions and matrix composition could explain the detected differences in the inactivation rates of *L. rhamnosus* GG. More specifically, whilst both systems will be in the rubbery state at room temperature, inulin based films were in the glassy state when stored at chilled conditions whereas the polydextrose systems were not. A similar behaviour was also observed in our recent work on spray dried powders containing soluble fibres (Yonekura et al., 2014). In this study it was shown that selecting a material that can provide a global protection against the sub-lethal effects of drying and storage conditions and including materials that can promote thermo-protection of bacterial cells do not necessarily shield probiotics upon storage and conversely. On the other hand, calculating the glass transition of the systems containing only gelatine as biopolymer we obtained a value of *T_g_* = 18.1 °C which implies that physical state is not the only factor that governs the *L. rhamnosus* GG lethality, and other factors such as the presence of an energetic substrate for probiotic cells may also be important. Thus, with appropriate selection the presence of prebiotic fibre can be a positive co-component for functionalised polymeric edible films.

## 4 Conclusions

The incorporation of prebiotic fibres on probiotic edible films exerts several beneficial effects to both the microstructure and the storage stability of immobilised probiotic cells. Notwithstanding some minor differences, prebiotics contribute to the increase of the matrix compactness and the reduction of porous and reticular structure detected in the case of control systems. In this study the stability of *L. rhamnosus* GG during the evaporation – drying film forming process was found to be fibre-dependent with glucose-oligosaccharides and polydextrose enhancing probiotic viability. Storage of the plasticised matrices under chilled and room temperature conditions led to a detectable reduction of the viable counts of *L. rhamnosus* GG with systems supplemented with inulin or wheat dextrin having greatest stability. However, in all cases the presence of the tested prebiotics was accompanied either by no change or an enhancement in the storage stability of the embedded living cells. Further investigation to unveil the physicochemical or biochemical phenomena that influence the stability of probiotic cells immobilised in plasticised biopolymer matrices is currently being carried out.

## Figures and Tables

**Table 1:** Physicochemical, optical and colour properties of edible films containing *L. rhamnosus* GG and different types of prebiotic fibres.

Edible film	Water activity *a_w_*	Residual water content† (g/100 g of film)	Thickness (mm)	Opacity	*L*^∗^	*a*^∗^	*b*^∗^	Δ*E*^∗^
Control	0.48 ± 0.01^a^	9.77 ± 0.23^a^	0.142 ± 0.02^a^	0.490 ± 0.07^a^	87.3 ± 0.9^a^	−1.44 ± 0.15^a^	8.9 ± 0.2^b^	–
Wheat dextrin	0.46 ± 0.02^a^	9.18 ± 0.47^a^	0.144 ± 0.03^a^	0.938 ± 0.06^c^	85.6 ± 0.7^a^	−2.01 ± 0.19^b^	10.4 ± 0.2^c^	2.23 ± 0.11^d^
Inulin	0.46 ± 0.02^a^	9.87 ± 0.18^a^	0.145 ± 0.01^a^	0.602 ± 0.03^b^	86.2 ± 0.2^a^	−1.83 ± 0.09^b^	9.9 ± 0.1^c^	1.59 ± 0.09^c^
Gluco-oligosaccharides	0.48 ± 0.02^a^	11.2 ± 0.34^b^	0.141 ± 0.04^a^	0.629 ± 0.04^b^	86.9 ± 0.8^a^	−1.60 ± 0.12^a^	7.8 ± 0.3^a^	1.19 ± 0.13^b^
Polydextrose	0.48 ± 0.01^a^	11.7 ± 0.23^b^	0.140 ± 0.01^a^	0.808 ± 0.05^c^	87.0 ± 0.5^a^	−1.55 ± 0.10^a^	8.4 ± 0.1^a^^,^^b^	0.58 ± 0.05^a^

a–d Different letter between rows indicate significantly different values (*p* < 0.05) according to Duncan’s post hoc means comparison test. Data are presented as mean ± SD (*n* = 3).

† Data refer to films conditioned at 54% RH.

**Table 2:** Inactivation rates of *L. rhamnosus* GG immobilised in plasticised gelatine matrices containing prebiotics and stored either under chilled or room temperature conditions.

Edible film	*k* 4 °C (% day^−1^)	Estimated shelf-life⁎ at 4 °C	*R*^2^	*k* 25 °C (%/day^−1^)	Estimated shelf-life at 25 °C	*R*^2^
Control	1.34 ± 0.03^c^	67	0.996	5.32 ± 0.04^c^	17	0.989
Wheat dextrin	1.11 ± 0.02^b^	81	0.988	3.33 ± 0.07^a^	27	0.959
Inulin	0.90 ± 0.04^a^	100	0.987	3.00 ± 0.12^a^	30	0.971
Gluco-oligosaccharides	1.44 ± 0.03^c^	63	0.951	3.98 ± 0.09^b^	23	0.988
Polydextrose	1.08 ± 0.03^b^	83	0.950	5.09 ± 0.11^c^	18	0.999

⁎ Shelf-life refers to the time required to induce the fate of the 90% of total viable cells of *L. rhamnosus* GG.

a–c Different letter between rows indicate significantly different values (*p* < 0.05) according to Duncan’s post hoc means comparison test. Data are presented as mean ± SD (*n* = 3).
